# Preliminary data on medication response to Suicide Crisis Syndrome

**DOI:** 10.1192/j.eurpsy.2025.1802

**Published:** 2025-08-26

**Authors:** A. Bassi De Toni, E. Wheeler, R. El Hayek, B. D. Park, M. Pompili, I. Galynker

**Affiliations:** 1 Psychiatry Residency Training Program, Faculty of Medicine and Psychology, Sapienza University of Rome, Roma, Italy; 2 Department of Psychiatry, Icahn School of Medicine at Mount Sinai, New York City , United States; 3 Department of Neurosciences, Mental Health and Sensory Organs, Faculty of Medicine and Psychology, Sant’Andrea Hospital, Sapienza University of Rome, Rome, Italy, Roma, Italy

## Abstract

**Introduction:**

Suicide Crisis Syndrome (SCS) is an acute suicidal state characterized by feelings of entrapment, affective disturbances, loss of cognitive control, hyperarousal, and social withdrawal. It has been demonstrated that SCS predicted suicidal behavior post discharge and that could help identifying high risk patients (Galynker I. Oxford University Press 2023; Yaseen ZS *et al.* Suicide Life Threat Behav Aug 2019;49(4):1124-1135). Until today, there is no valid consensus on which medication class is more effective in reducing the acute symptoms of SCS, which often accompany suicidal patients.

**Objectives:**

The present study aims to evaluate medication response to SCS along hospitalization into a psychiatric unit and to determine if certain drug classes can alleviate SCS symptoms.

**Methods:**

The sample consisted of 133 participants, aged 18 to 85, recruited across four Mount Sinai Health System hospitals in New York City, USA and admitted to the psychiatric units for suicidal behavior in the past week. Patients were assessed at intake and discharge with the Suicide Crisis Syndrome -Checklist (SCS-C) to evaluate the presence of SCS symptoms. Administered medication were divided into 5 classes: antipsychotics, antidepressants, mood stabilizers, benzodiazepines and opioids.

**Results:**

Looking at administered medications, descriptive analysis showed an overall decrease in mean SCS-C scores from intake to discharge with antipsychotics and opioids being associated with greater SCS symptoms reduction throughout hospitalization. On the other hand, participants taking antidepressants, mood stabilizers or benzodiazepines did not show different SCS-C results than participants who were not administered these medications.

**Conclusions:**

Our results are preliminary and to gain a clearer perspective a larger sample size would be needed. Nonetheless, antipsychotics drugs given their broad psychopharmacological properties might be able to address the difficult and heterogeneous symptomatology of suicidal patients, especially loss of cognitive control aspect of the SCS. Given that opioids are used to treat emotional pain, they may be an effective treatment for symptoms of SCS as well. These results highlight the need for further studies and randomized clinical trials to adequately assess the effectiveness and safety of these medications in treating SCS.

**Disclosure of Interest:**

None Declared

